# Identification of deleterious variants in patients with male infertility due to idiopathic non-obstructive azoospermia

**DOI:** 10.1186/s12958-022-00936-z

**Published:** 2022-04-02

**Authors:** Dongdong Tang, Kuokuo Li, Hao Geng, Chuan Xu, Mingrong Lv, Yang Gao, Guanxiong Wang, Hui Yu, Zhongmei Shao, Qunshan Shen, Hui Jiang, Xiansheng Zhang, Xiaojin He, Yunxia Cao

**Affiliations:** 1 Department of Obstetrics and Gynecology, Reproductive Medicine Center, the First Affiliated Hospital of Anhui Medical University, No 218 Jixi Road, Hefei, 230022 Anhui China; 2 NHC Key Laboratory of Study On Abnormal Gametes and Reproductive Tract (Anhui Medical University), No 81 Meishan Road, Hefei, 230032 Anhui China; 3 Key Laboratory of Population Health Across Life Cycle (Anhui Medical University), Ministry of Education of the People’s Republic of China, No. 81 Meishan Road, Hefei, 230032 Anhui China; 4 Reproductive Medicine Center, Peking University Third Hospital, No 38 Xueyuan Road, Beijing, 100191 China; 5 Department of Urology, the First Affiliated Hospital of Anhui Medical University, No 218 Jixi Road, Hefei, 230022 Anhui China; 6 Anhui Provincial Human Sperm Bank, The First Affiliated Hospital of Anhui Medical University, Hefei, 230022 China

**Keywords:** Male infertility, Non-obstructive azoospermia, Gene, Variant

## Abstract

**Background:**

Non-obstructive azoospermia (NOA) is the most severe type of male infertility, affecting 1% of men worldwide. Most of its etiologies remain idiopathic. Although genetic studies have identified dozens of NOA genes, monogenic mutations can also account for a small proportion of idiopathic NOA cases. Hence, this genetic study was conducted to explore the causes of monogenic variants of NOA in a cohort of Chinese patients.

**Methods:**

Following the screening using chromosomal karyotyping, Y chromosome microdeletion analyses, and sex hormone assessments, subsequent whole-exome sequencing analysis was performed in 55 unrelated idiopathic NOA patients with male infertility to explore potential deleterious variants associated with spermatogenesis. We also performed Sanger sequencing to demonstrate the variants. Testicular biopsy or microsurgical testicular sperm extraction was also performed to confirm the diagnosis of NOA and identify spermatozoa. Hematoxylin and eosin staining was performed to assess the histopathology of spermatogenesis.

**Results:**

Abnormal testicular pathological phenotypes included Sertoli cell-only syndrome, maturation arrest, and hypospermatogenesis. Using bioinformatics analysis, we detected novel variants in two recessive genes, *FANCA* (NM_000135, c.3263C > T, c.1729C > G) and *SYCE1* (NM_001143763, c.689_690del); one X-linked gene, *TEX11* (NM_031276, c.466A > G, c.559_560del); and two dominant genes, *DMRT1* (NM_021951, c.425C > T, c.340G > A) and *PLK4* (NM_001190799, c.2785A > G), in eight patients, which corresponded to 14.55% (8/55) of the patients.

**Conclusion:**

This study presented some novel variants of known pathogenic genes for NOA. Further, it expanded the variant spectrum of NOA patients, which might advance clinical genetic counseling in the future.

**Supplementary Information:**

The online version contains supplementary material available at 10.1186/s12958-022-00936-z.

## Background

Approximately one in six couples face infertility, with male factors accounting for 50% of all factors. A prevalence study found that 1% of men and 10%–20% of male infertility cases were diagnosed with azoospermia, which seriously influence health worldwide. Non-obstructive azoospermia (NOA), characterized by quantitative impairment of spermatogenesis, including three testicular pathological phenotypes (Sertoli cell-only syndrome [SCOS], maturation arrest [MA], and hypospermatogenesis), is the most severe form of male infertility [1, 2]. The developed micro-dissection testicular sperm extraction and intracytoplasmic sperm injection can help a small number of NOA patients to obtain their biological offspring. In contrast, this pathogenic genetic risk might also be transmitted to the next male generations who confront infertility.

With the development of next-generation sequencing technologies, genetic factor disruptions play an important role in the formation of NOA cases. Recent studies identified putative pathogenic variants in approximately 38 candidate genes using whole-exome sequencing (WES) or whole-genome sequencing [2]. For example, pathogenic variants in *MEIOB* have been detected in NOA patients from both consanguineous and non-consanguineous families [3, 4]. Because of its indispensable role in homologous recombination in meiosis I, *MEIOB* can also affect female patients with primary ovarian insufficiency [5]. Despite great advances in the genetic findings of NOA, the pathogenic genetic mechanism of a large proportion of patients is still unknown. Moreover, some candidate genes exhibited weak genetic evidence because of the lack of recurrent studies carrying pathogenic variants in the same genes. Large cohort WES of NOA patients was necessary to perform genotype–phenotype analysis.

In this study, we aimed to expand the variant spectrum of known candidate genes of NOA. The 55 NOA patients with testicular pathological phenotypes of SCOS, MA, and hypospermatogenesis were sequenced using WES. We detected putative pathogenic variants in five candidate genes with matched inheritance patterns, including *FANCA*, *SYCE1*, *TEX11*, *DMRT1*, and *PLK4*.

## Methods

### Sample collection

A total of 55 patients with NOA and infertility were recruited from the First Affiliated Hospital of Anhui Medical University. The couples did not achieve a clinical pregnancy during more than 12 months of unprotected intercourse. Idiopathic NOA was diagnosed by careful medical history, physical examination, laboratory examination, imaging, and testicular biopsy. All patients excluded other risk factors of infertility/NOA, including chromosomal abnormalities, Y chromosome microdeletion, cryptorchidism, radiotherapy and chemotherapy, viral or bacterial orchitis, epididymitis, epididymo-orchitis, undescended testis and hypogonadism as well as sexually transmitted infections [6, 7]. We collected whole-peripheral blood samples from patients and performed WES as described in our previous study [8]. This research was approved by the Ethics Committee of the First Affiliated Hospital of Anhui Medical University, and all patients agreed to participate and signed the informed consent form.

### Semen analysis

Based on the World Health Organization guidelines (5th edition), semen sample was collected and examined. Semen samples were collected following 3–7 days of abstinence and evaluated after liquefaction for 30 min at 37 °C. Centrifugation was performed at 3000 g to detect sperm by microscopic examination of semen when no sperm were found by routine test [9]. To confirm the clinical diagnosis, we detected sperm at three different times with an interval of more than 2 weeks.

### Genetic risk factors identification by using WES and Sanger sequencing

The whole-exome was sequenced using the Illumina HiSeq platform. The original FASTQ data were mapped to the human genome using BWA software, and SAMtools and GATK were used to call genetic variants. We annotated variants into the allele frequency database (1000G, EXAC03_EAS, gnomAD_exome_EAS), deleterious prediction tools (SIFT, PolyPhen-2, Mutation Taster, and CADD), and Human Gene Mutation Database (HGMD) using ANNOVAR [10] and dbNSFP [11]. Common variants with allele frequency of > 0.05 were excluded. We focused on loss-of-function (including splicing (≤ 2 bp), stopgain, stoploss, and frameshift indels) and deleterious missense variants. The variant that was predicted to be deleterious by more than three of four software, including SIFT, PolyPhen-2, Mutation Taster, and CADD (score > 20), were defined as a deleterious variant. Moreover, variants defined as deleterious in HGMD were included in the analysis. Sanger sequencing was performed to validate the inheritance pattern of the putative pathogenic variants.

### Hematoxylin and eosin (H&E) staining of testicular tissue

We performed H&E staining to detect the detailed histopathology of the testis in NOA patients. Testicular tissue was fixed with Bouin solution for more than 24 h. Next, gradient alcohol (75%, 85%, 90%, 95%, and 100%) was used to dehydrate the fixed testicular tissue, embedded in paraffin, and sectioned into 3-μm-thick sections. Then, H&E staining was performed to stain the nucleus and cytoplasm.

## Results

### Bi-allelic pathogenic variants of two recessive genes FANCA and SYCE1 in three NOA patients

**Fig. 1:**
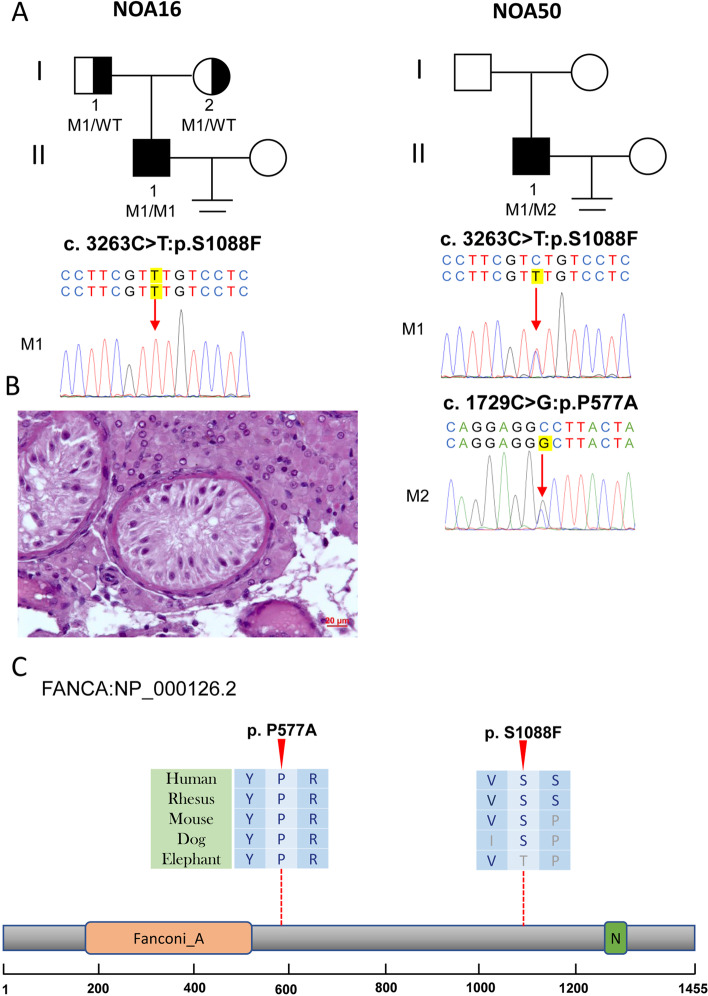
Variants of *FANCA* in NOA16 and NOA50. **A** The families affected by the variants in *FANCA*. The red dotted lines indicate mutated positions in the Sanger sequencing results. **B** Testicular histopathology of NOA16. **C** The mutated positions of *FANCA* are conserved among species (red arrows). And the dotted lines indicate the positions of the *FANCA* variant in the *FANCA* protein. M, mutation; WT, wild type

NOA16 was diagnosed with infertility for 2 years with non-contraceptive intercourse. We found no sperm in recurrent semen analysis of NOA16, and physical examination showed typical development of secondary sexual characteristics, normal epididymis, vas deferens, and testicular size. Hormone tests revealed an abnormally increased level of follicle-stimulating hormone (FSH) (Table 1). We performed a testicular biopsy and subsequent H&E staining of NOA16 and found a testicular pathological phenotype of SCOS (Fig. 1). NOA16 was carrying homozygous missense variants in *FANCA* (NM_000135: c.3263C > T; p.S1088F) with allele frequency < 0.05, in three databases (Table 1). In addition, three of the four tools (SIFT, PolyPhen-2, MutationTaster, CADD) predicted that this missense mutation was deleterious. Sanger sequencing confirmed that the parents were heterozygous carriers. Additionally, another patient, NOA50, exhibited potential compound heterozygous *FANCA*. One heterozygous variant was consistent with that in NOA16, and the other variant (NM_000135: c.1729C > G; p.P577A) was extremely rare (0, 0, 0) in the three allele frequency databases (Table 1). Four studies generally predicted that this variant was deleterious. The sex hormones of NOA50 also showed an abnormally increased level of FSH (Table 1). Unfortunately, we were unable to perform a testicular biopsy and validate these two variants based on Sanger sequencing due to the disagreement of NOA50.

**Table 1:** Deleterious variants detected in patients with non-obstructive azoospermia and related clinical phenotypes

Individual	NOA16	NOA50	NOA51	NOA22	NOA25	NOA42	NOA39	NOA49
Gene	*FANCA*	*FANCA*	*SYCE1*	*DMRT1*	*DMRT1*	*PLK4*	*TEX11*	*TEX11*
Inheritance pattern	AR	AR	AR	AD	AD	AD	X-linked	X-linked
RefSeq accession number	NM_000135	NM_000135	NM_001143763	NM_021951	NM_021951	NM_001190799	NM_031276	NM_031276
Age	27	27	31	27	31	29	32	25
Secondary sexual characteristics	Normal	Normal	Normal	Normal	Normal	Normal	Normal	Normal
testicular volume(Left/Right, ml)	8/8	8/8	15/15	10/10	10/10	12/12	12/12	10/10
Somatic karyotype	46,XY	46,XY	46,XY	46,XY	46,XY	46,XY	46,XY	46,XY
Y Chromosome microdeletions	No	No	No	No	No	No	No	No
Follicle-stimulating hormone (IU/L)	23.87	23.87	3.85	16.32	26.54	29.24	8.44	4.02
Luteinizing hormone (IU/L)	6.1	6.10	0.41	6.44	11.35	7.05	6.33	5.33
Testosterone (nmol/L)	14.03	14.03	31.14	17.95	7.07	10.75	10.75	13.34
Estradiol (pmol/L)	NA	90	372	241	23	73	97	132
Prolactin (ng/ml)	NA	8.26	14.6	11.88	10.37	8.11	8.92	10.24
Testis histology	SCOS	ND	MA	SCOS	SCOS	SCOS	Hypospermatogenesis	MA
Hom/Het	Hom	Het/ Het	Hom	Het	Het	Het	Hemi	Hemi
cDNA mutation	c.3263C > T	c.3263C > T/ c.1729C > G	c.689_690del	c.425C > T	c.340G > A	c.2785A > G	c.466A > G	c.559_560del
Mutation type	Missence	Missence/ Missense	Frameshift	Missense	Missense	Missense	Missense	Frameshift
Protein alteration	p.S1088F	p.S1088F/ p.P577A	p.F230fs	p.A142V	p.V114M	p.M929V	p.M156V	p.M187fs
1KGP	0.0218	0.0218/ 0	0	0	0	0	0	0
EXAC_EAS	0.0235	0.0235/ 0	0	0	0	0	0.0039	0
gnomAD_EAS	0.0265	0.0265/ 0	0.0001	0	0	0	0.0034	0
SIFT	D	D/ D	NA	T	D	D	T	NA
PolyPhen-2	P	P/ D	NA	D	D	P	B	NA
MutationTaster	N	N/ D	NA	D	D	D	N	NA
CADD	21.8	21.8/ 23.9	NA	22.2	33	23.9	22.2	NA
HGMD	NA	NA/ NA	NA	NA	NA	NA	D	NA
Validation in patient	Yes	Yes/ Yes	Yes	Yes	Yes	Yes	Yes	Yes
Mother/Father genotype	Het/Het	ND/ ND	ND/Het	ND	ND	ND	Het/WT	ND

*AR* autosomal recessive, *AD* autosomal dominant, *1KGP* 1000 Genomes Project, *ExAc_EAS* the data of East Asian in Exome Aggregation Consortium, *gnomAD_EAS* the data of East Asian in the Genome Aggregation Database, *D* Damaging, *T* Tolerant, *P* Possibly Damaging, *B* Benign, *N* Polymorphism, *ND* Not Detect, *SCOS* Sertoli cell only syndrome, *MA* maturation arrest

**Fig. 2:**
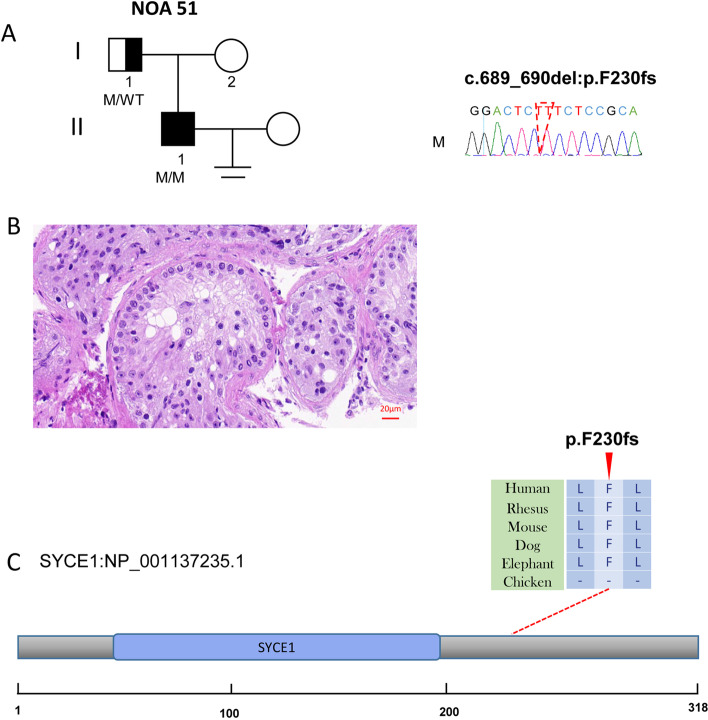
The variant of *SYCE1* in NOA51. **A** The family affected by the variant in *SYCE1*. The red dotted line indicates the mutated position in the Sanger sequencing. **B** Testicular histopathology. **C** The mutated position of *SYCE1* is conserved among species (red arrows). And the dotted line indicates the position of *the SYCE1* variant in SYCE1 protein. M, mutation; WT, wild type

NOA51 was found to carry a homozygous frameshift variants in *SYCE1* (NM_001143763, c.689_690del; p.F230fs) with extremely rare allele frequencies (0, 0, 0.0001) in three databases (Table 1). The levels of hormones in NOA51 were within the normal range (Table 1). We performed a testicular biopsy with the agreement of NOA51 and found the phenotype of MA (Fig. 2). Sanger sequencing confirmed the homozygous and heterozygous carriers in NOA51 and his biological father.

### Heterozygous variants of dominant gene DMRT1 and PLK4 in three NOA patients

**Fig. 3:**
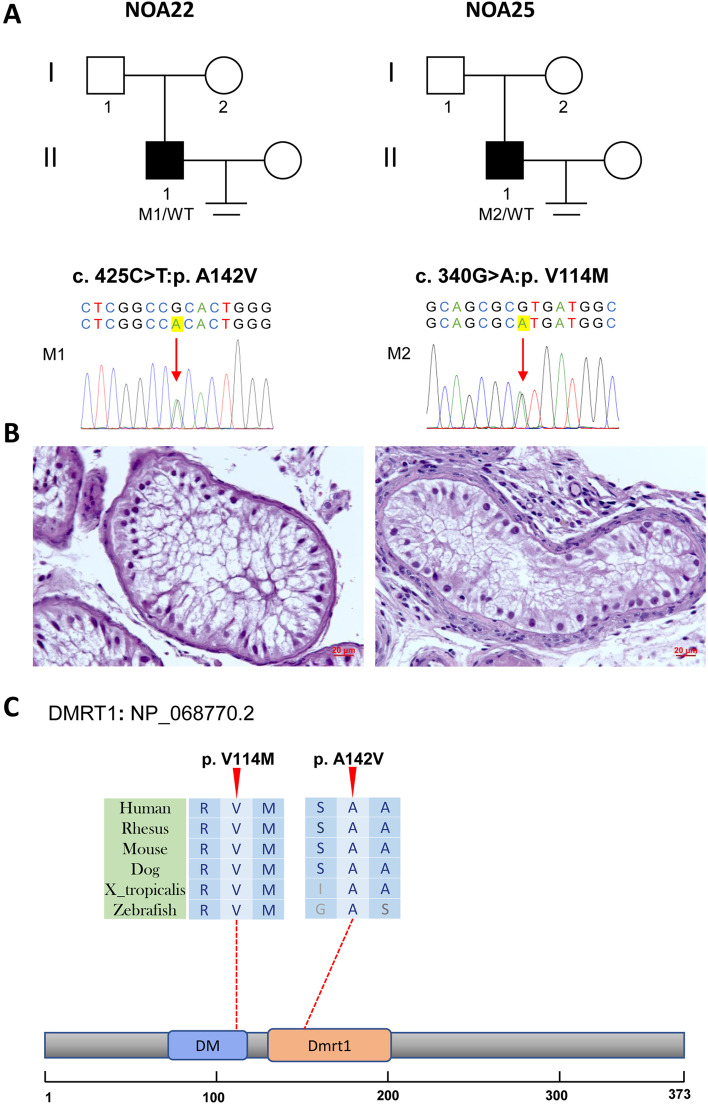
Variants of *DMRT1* in NOA22 and NOA25. **A** The families affected by the variants in *DMRT1*. The red arrows indicate mutated positions in the Sanger sequencing results. **B** Testicular histopathology. **C** The mutated positions of *DMRT1* are conserved among species (red arrows). And the dotted line indicates the position of *DMRT1* variants in *DMRT1* protein. M, mutation; WT, wild type

We found two NOA patients carrying heterozygous variants in *DMRT1* and related amino acids in the functional domain. NOA22 carries NM_021951:c.425C > T; p.A142V, which was extremely rare (0, 0, 0) in the three allele frequency databases (Table 1). Three of the four tools generally predicted that this variant was deleterious. Sanger sequencing was performed to validate the heterozygous state. The sex hormones of NOA22 also showed abnormally increased FSH value (Table 1). Based on the agreement of NOA22, we performed a testicular biopsy and found only Sertoli cells (Fig. 3). The other patient, NOA25 carrying NM_021951: c.340G > A; p.V114M, also exhibited extremely rare (0, 0, 0) in the three allele frequency databases (Table 1). Four general tools predicted that this variant would be deleterious. Sanger sequencing was performed to validate the heterozygous state. Abnormal hormones, including increased FSH levels, were found in NOA25 (Table 1). Based on the agreement of NOA25, we performed a testicular biopsy and found that NOA25 exhibited the pathological phenotype of SCOS (Fig. 3). In addition, after an exhaustive understanding of microsurgical testicular sperm extraction, NOA25 consented to undergo the procedure. This patient succeeded in achieving pregnancy using microsurgical testicular sperm extraction and intracytoplasmic sperm injection. Unfortunately, we were unable to validate these variants based on Sanger sequencing because of the disagreement of the offering DNA sample of parents.

**Fig. 4:**
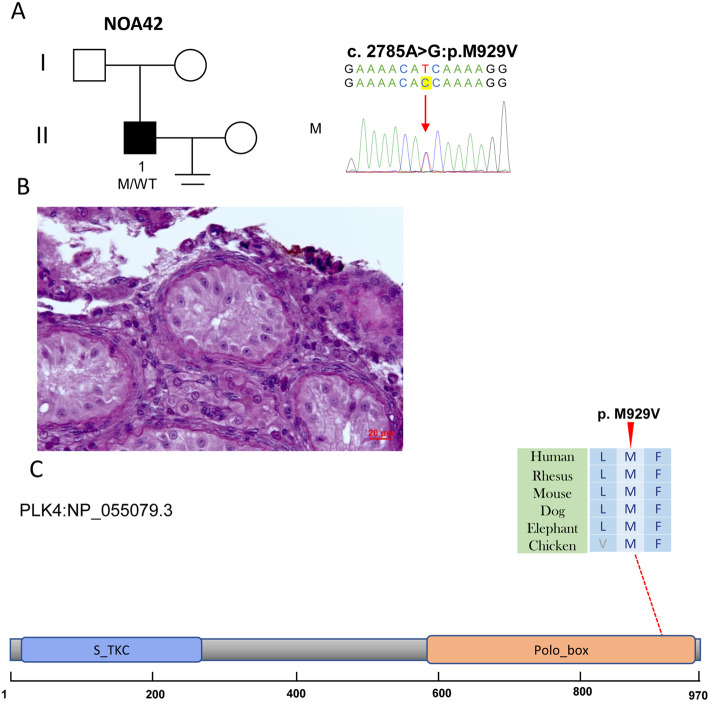
The variant of *PLK4* in NOA42. **A** The family affected by the variant in *PLK4*. The red arrow indicates the mutated position in the Sanger sequencing results. **B** Testicular histopathology. **C** The mutated position of *PLK4* is conserved among species (red arrows). And the dotted line indicates the position of *PLK4* variant in PLK4 protein. S_TKC, Serine/Threonine protein kinases, catalytic domain; M, mutation; WT, wild type

We also found that NOA42 carries a missense variant in the dominant *PLK4* gene (NM_001190799, c.2785A > G; p.M929V) (Table 1). All allele frequencies in the three databases were zero. Four tools generally predicted that this variant was deleterious. Sanger sequencing validated the heterozygous state of c.2785A > G. An abnormal hormone of the increased FSH value was found in NOA42 (Table 1). We performed testicular biopsy with the agreement of NOA22, and only found Sertoli cells (Fig. 4). However, we were unable to validate these variants based on Sanger sequencing because of the disagreement for DNA sample collection of parents.

### Hemizygous variants of TEX11 in two NOA patients

**Fig. 5:**
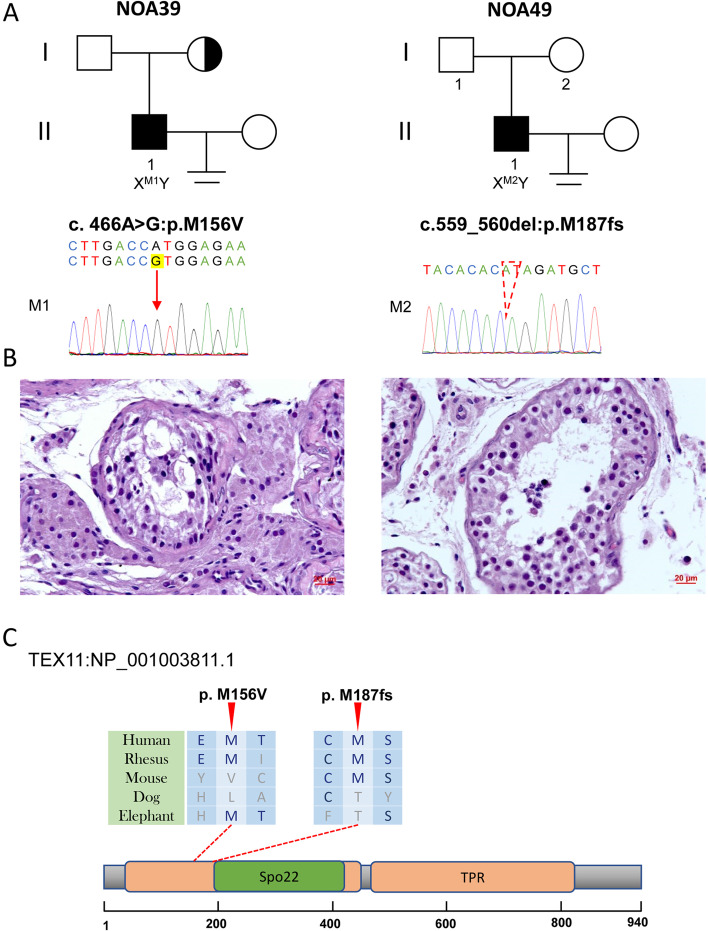
Variants of *TEX11* in NOA39 and NOA49. **A** The families affected by the variants in *TEX11*. The red dotted line indicates mutated positions in the Sanger sequencing results. **B** Testicular histopathology. **C** The mutated positions of *TEX11* are conserved among species (red arrows). And the dotted lines indicate the positions of *TEX11* variants in TEX11 protein. M, mutation; WT, wild type

*TEX11* is a well-known X-linked NOA pathogenic gene. This study identified two novel putatively pathogenic hemizygous variants in two patients with relatively different phenotypes (Table 1). NOA39 carries a missense variant (NM_031276:c.466A > G; p.M156V) with an allele frequency of < 0.01, which is inherited from his mother. The HGMD database defines this variant as being deleterious. In NOA39, the hormone levels were as follows: FSH 8.44 IU/L, LH 6.33 IU/L, T 10.75 nmol/L, estradiol 97 pmol/L, and prolactin 8.92 ng/ml. NOA39 showed hypospermatogenesis based on a testicular biopsy test (Fig. 5). The other patient, NOA49, carried a frameshift deletion NM_031276:c.559_560del, p.M187fs with zero allele frequency. The frameshift variant was a functional loss, resulting in severe structural and functional impairment of the TEX11 protein. In NOA49, the hormone levels were as follows: FSH 4.02 IU/L, LH 5.33 IU/L, T 13.34 nmol/L, estradiol 132 pmol/L, and prolactin 10.24 ng/ml. The phenotype of NOA49 was different from that of NOA39 and exhibited MA (Fig. 5). Sanger sequencing validated these two variants in patients. However, we cannot validate the inheritance pattern because of the disagreement of the offering DNA sample of parents.

## Discussion

Genetic factors contribute significantly to NOA patients, and high-throughput sequencing has provided unprecedented opportunities to decode risk genes or variants. Genetic studies identified several dozens of genes in patients with NOA. Still, the monogenic variants can also account for a small proportion of idiopathic NOA cases, and the genetic mechanism in most of the patients was unknown. We performed WES of 55 NOA patients to explore the genetic mechanism and advance the genetic spectrum for clinical diagnosis. We detected variants in five previously reported NOA genes in eight patients, including two recessive genes, *FANCA*, *SYCE1*, 1 X-linked gene *TEX11*, and two dominant genes *DMRT1*, *PLK4*. We summarized variants in previous studies and present study of these genes in Supplementary table 1.

FANCA is involved in interstrand cross-link repair or a cell cycle checkpoint. The bi-allelic variants in *FANCA* contribute to a large proportion of Fanconi anemia, an autosomal recessive disease characterized by progressive bone marrow failure [12, 13]. Recent studies found *FANCA* variants in patients with premature ovarian insufficiency (POI) [14] and NOA [15]. Kraus et al. first reported bi-allelic variants in two patients with SCOS; however, this result might not be used for clinical diagnosis without recurrent studies. Our study detected pathogenic *FANCA* variants in two additional patients with NOA, which increased the clinical evidence.

SYCE1 is a protein component of the synaptonemal complex during meiosis. Bi-allelic variants in *SYCE1* were also detected in patients with POI [16, 17] and NOA [18, 19], suggesting an indispensable role for SYCE1 in meiosis and germ cell development. The previously identified two splicing variants (NM_001143763: c.197-2A > G, c.375-2A > G) [18, 19] result in a truncated product of amino acids in the structural core of SYCE1 (amino acids position: 25–79), which bind to the N-terminus of SIX6OS1 and form a synaptonemal complex [20]. The variant in our study resulted in a frameshift at position 230 that locates in the second interface to bind with the downstream sequence within SIX6OS1 1–262, which is similar to NM_001143763: c.613C > T that was identified in patients with POI [17, 20].

DMRT1 is a transcription factor that plays a role in male sex determination and differentiation by controlling testis development and male germ cell proliferation [21]. The abnormal testicular pathological phenotypes of patients carrying *DMRT1* variants are heterogeneous and characterized by SCOS, MA, spermatogonial arrest, and spermatocyte arrest [22, 23]. We found two patients with SCOS carrying two heterozygous missense mutations located in the functional domain (DNA-binding domain and double-sex/mab3-related transcription factor 1). Our study might increase the clinical evidence *of DMRT1* from moderate from a previous study to strong [24].

PLK4 is a regulator of centriole biogenesis and plays an important role in cell division [25, 26]. A previous study found that homozygous loss-of-function variants in *PLK4* contribute to the formation of microcephaly and chorioretinopathy [26]. However, Harris et al. found that heterozygous variants in *PLK4* resulted in hypogonadism and germ cell loss in mice [27]. Miyamoto et al. first reported a heterozygous frameshift variant located in serine/threonine protein kinases, the catalytic domain of PLK4 in a patient with SCOS [28]. We found a heterozygous missense variant located in the polo-box domain of PLK4 in a patient with SCOS, and the mechanism of this variant should be further validated by functional assays. Moreover, although we provided recurrent variants, the clinical evidence of PLK4 for NOA is limited, and more pathogenic variants are required to research clinical significance [29, 30].

*TEX11* is an X-linked testis-specific gene involved in meiotic recombination and chromosomal synapsis and is defined as a strong clinical evidence gene for NOA [31–34]. Most of the testicular phenotypes of patients carrying the *TEX11* variants were MA. Our study found one frameshift *TEX11* variant in patients with the MA phenotype. Another patient carrying the missense *TEX11* variant exhibited the phenotype of hypospermatogenesis. This variant was already detected in patients with MA in a previous study, which suggested phenotypic heterogeneity [33].

We detected putative pathogenic variants in five candidate genes with matched inheritance patterns, including *FANCA*, *SYCE1*, *TEX11*, *DMRT1*, and *PLK4* in eight patients, which corresponded to 14.55% (8/55) of the patients. No candidate pathogenic genes were found in the other 47 patients with iNOA. Some possible reasons may account for this result. First, some nongenetic etiologies may also lead to spermatogenic failure in these patients. Second, only WES was performed in this present study, copy number variations and structural variations in some genes involved in spermatogenesis can also cause NOA, whereas they were not performed in our study. Third, some regulatory elements in noncoding and intergenic regions may affect expressions of some NOA-related genes. The pathogenic variants located in these regions may lead to formation of NOA.

There are two limitations to this study. First, we only obtained the DNA information of these patients with NOA and used bioinformatics tools to predict the deleterious missense variants. Further experiments on functional level is necessary to assess the pathogenicity of the variants. Second, although they matched the genotype and phenotype of these genes, we could not validate all inherited models due to unavailable DNA samples from the parents of the patients.

## Conclusions

Taken together, we performed WES analysis of 55 NOA patients and detected novel pathogenic variants in five known NOA candidate genes in 14.55% of patients with NOA. Our results widened the genetic spectrum and provide the opportunity for more accurate genetic diagnostics of NOA.

## Supplementary Information

**Additional file 1: Table S1.** Variants in previous studies and this present study of FANCA, SYCE1, DMRT1, PLK4 and TEX11.

## Data Availability

The datasets used and/or analyzed during the current study are available from the corresponding author upon reasonable request.

## Abbreviations

NOA: Non-obstructive azoospermia
WES: Whole-exome sequencing
H&E: Hematoxylin and eosin staining
POI: Premature ovarian insufficiency
SCOS: Sertoli cell-only syndrome
MA: Maturation arrest
