# Correction: Identification of deleterious variants in patients with male infertility due to idiopathic non-obstructive azoospermia

**DOI:** 10.1186/s12958-022-00950-1

**Published:** 2022-05-20

**Authors:** Dongdong Tang, Kuokuo Li, Hao Geng, Chuan Xu, Mingrong Lv, Yang Gao, Guanxiong Wang, Hui Yu, Zhongmei Shao, Qunshan Shen, Hui Jiang, Xiansheng Zhang, Xiaojin He, Yunxia Cao

**Affiliations:** 1 Department of Obstetrics and Gynecology, Reproductive Medicine Center, the First Affiliated Hospital of Anhui Medical University, No 218 Jixi Road, Hefei, 230022 Anhui China; 2 NHC Key Laboratory of Study On Abnormal Gametes and Reproductive Tract (Anhui Medical University), No 81 Meishan Road, Hefei, 230032 Anhui China; 3 Key Laboratory of Population Health Across Life Cycle (Anhui Medical University), Ministry of Education of the People’s Republic of China, No. 81 Meishan Road, Hefei, 230032 Anhui China; 4 Reproductive Medicine Center, Peking University Third Hospital, No 38 Xueyuan Road, Beijing, 100191 China; 5 Department of Urology, the First Affiliated Hospital of Anhui Medical University, No 218 Jixi Road, Hefei, 230022 Anhui China; 6 Anhui Provincial Human Sperm Bank, The First Affiliated Hospital of Anhui Medical University, Hefei, 230022 China

**Correction: Reprod Biol Endocrinol 20, 63 (2022)**

**https://doi.org/10.1186/s12958-022-00936-z**

Following the publication of the original article [1], it was noted that due to a typesetting error the figure images for Figures 1-5 in the PDF version were not updated and an error was found in Table 1.

The correct Figs. 1, 2, 3, 4, 5 and Table 1 are shown below.

**Fig. 1:**
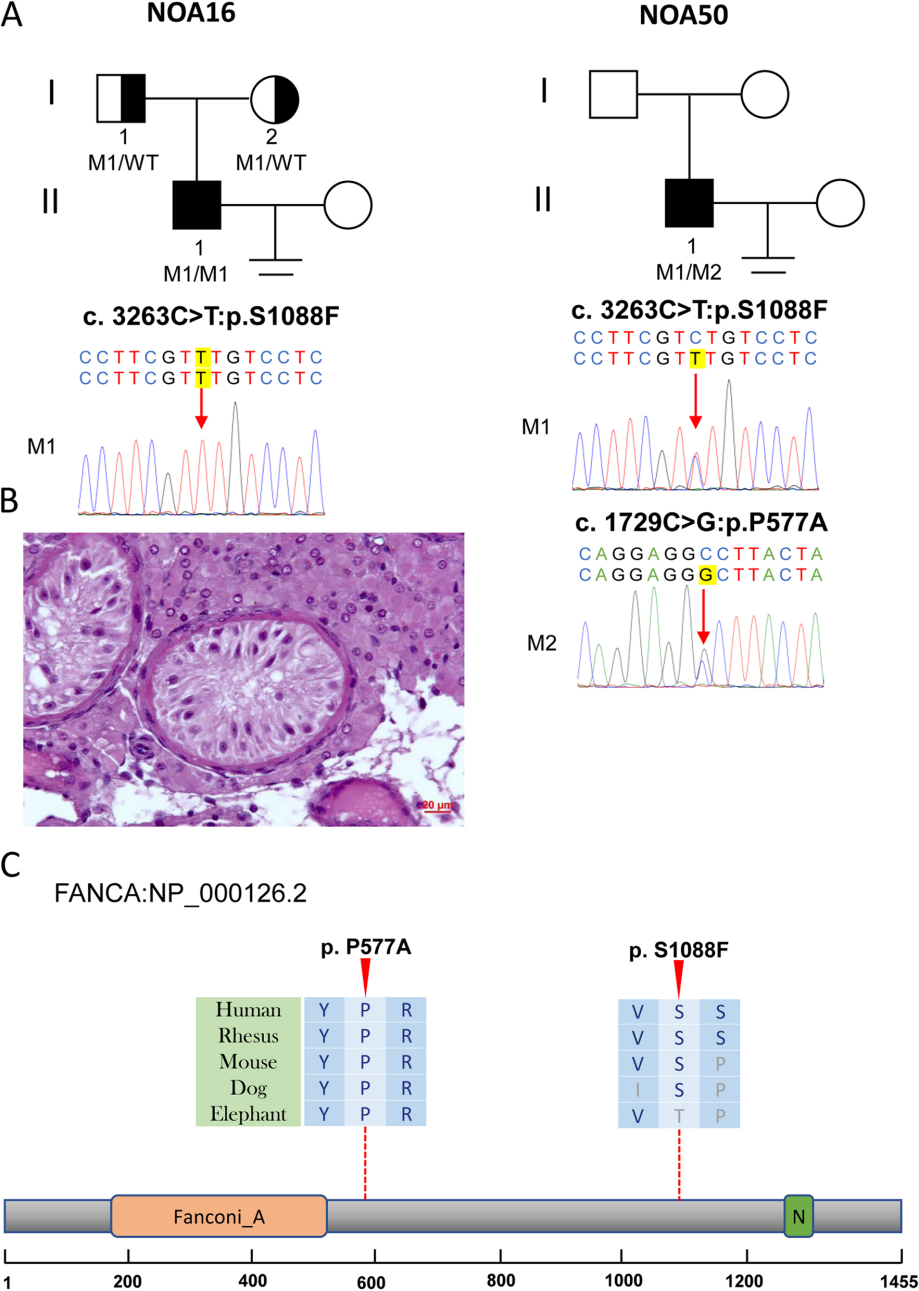
Variants of *FANCA* in NOA16 and NOA50. **A** The families affected by the variants in *FANCA*. The red dotted lines indicate mutated positions in the Sanger sequencing results. **B** Testicular histopathology of NOA16. **C** The mutated positions of *FANCA* are conserved among species (red arrows). And the dotted lines indicate the positions of the *FANCA* variant in the *FANCA* protein. M, mutation; WT, wild type

**Fig. 2:**
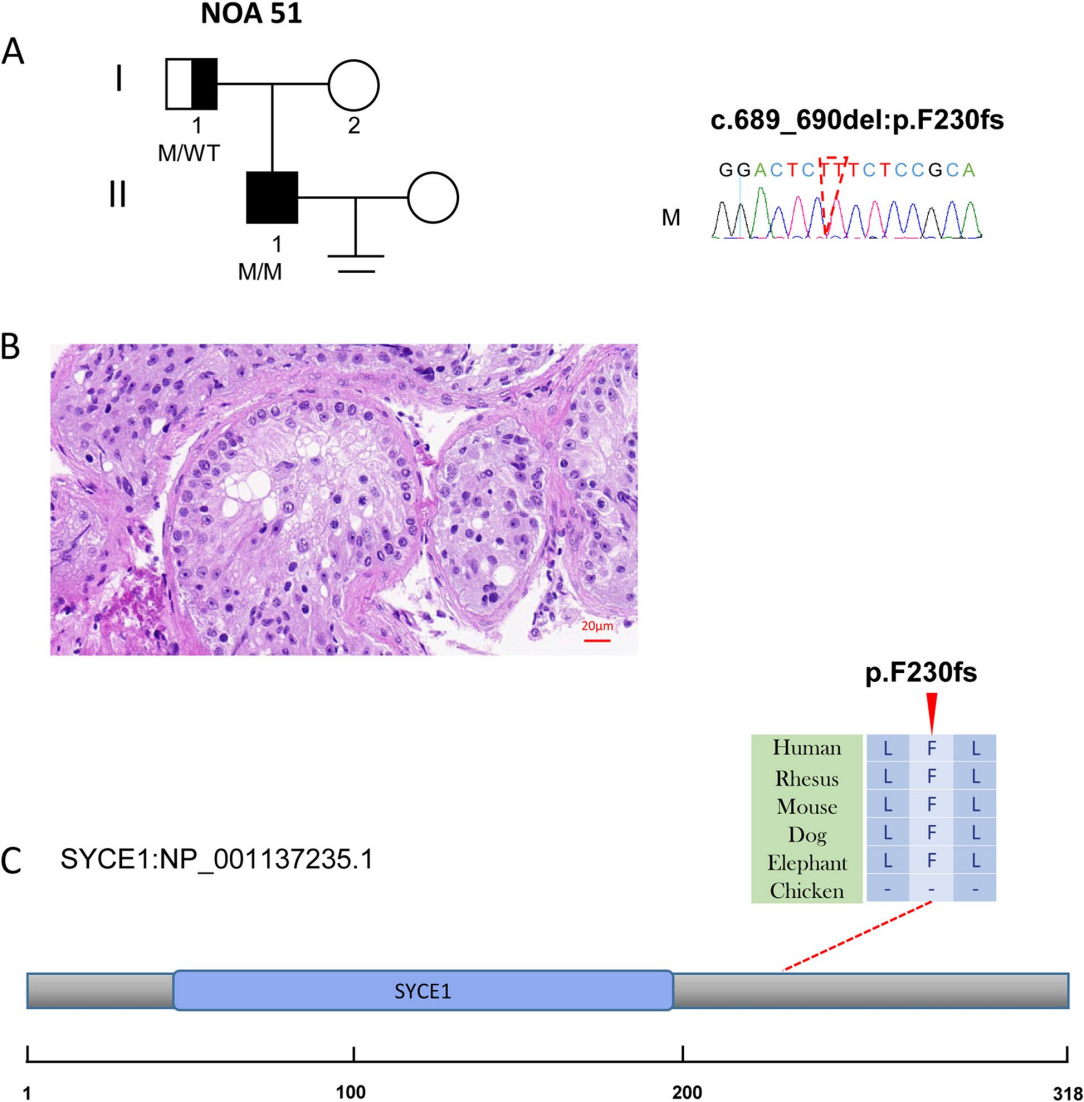
The variant of *SYCE1* in NOA51. **A** The family affected by the variant in *SYCE1*. The red dotted line indicates the mutated position in the Sanger sequencing. **B** Testicular histopathology. **C** The mutated position of *SYCE1* is conserved among species (red arrows). And the dotted line indicates the position of *the SYCE1* variant in SYCE1 protein. M, mutation; WT, wild type

**Fig. 3:**
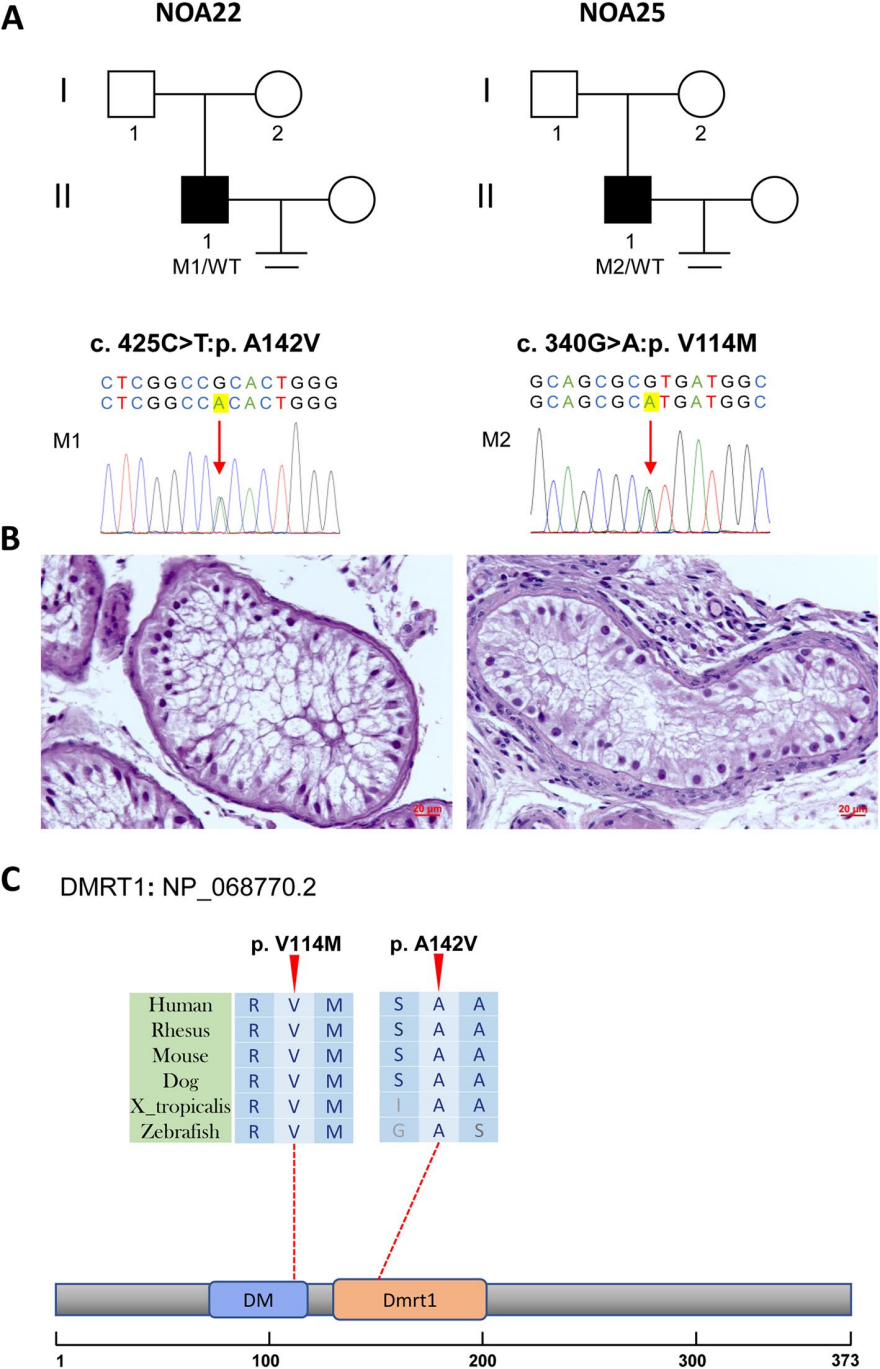
Variants of *DMRT1* in NOA22 and NOA25. **A** The families affected by the variants in *DMRT1*. The red arrows indicate mutated positions in the Sanger sequencing results. **B** Testicular histopathology. **C** The mutated positions of *DMRT1* are conserved among species (red arrows). And the dotted line indicates the position of *DMRT1* variants in *DMRT1* protein. M, mutation; WT, wild type

**Fig. 4:**
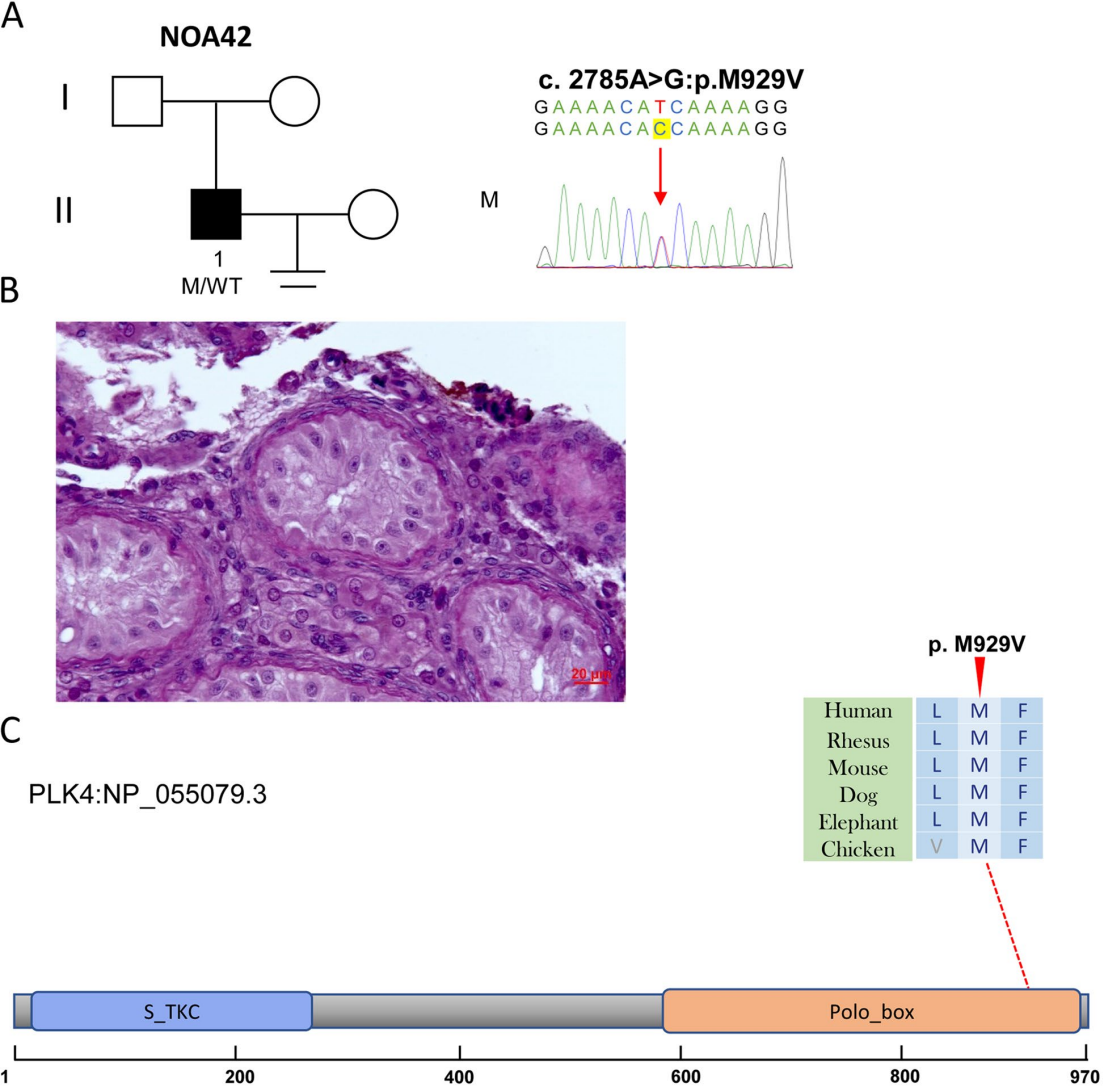
The variant of *PLK4* in NOA42. **A** The family affected by the variant in *PLK4*. The red arrow indicates the mutated position in the Sanger sequencing results. **B** Testicular histopathology. **C** The mutated position of *PLK4* is conserved among species (red arrows). And the dotted line indicates the position of *PLK4* variant in PLK4 protein. S_TKC, Serine/Threonine protein kinases, catalytic domain; M, mutation; WT, wild type

**Fig. 5:**
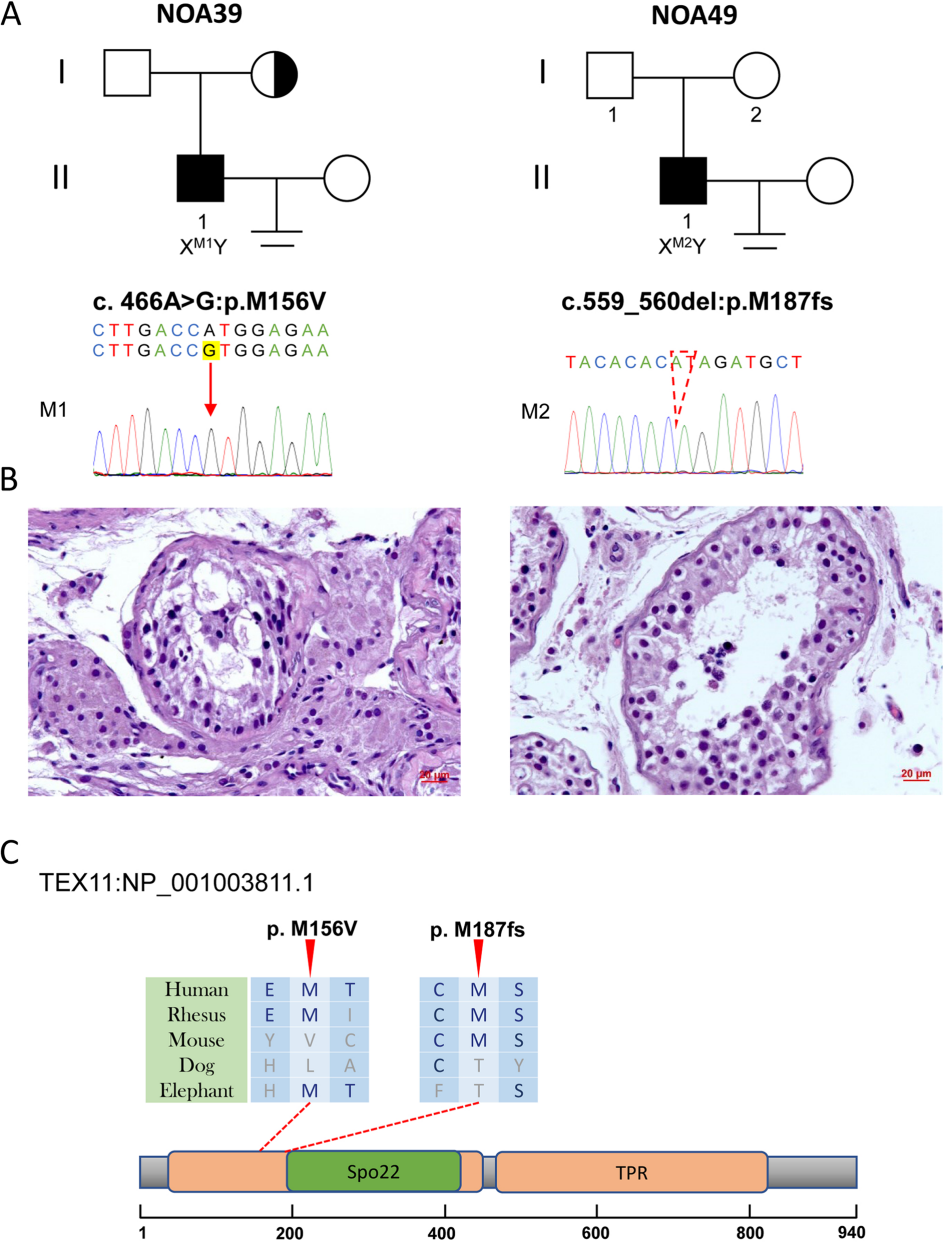
Variants of *TEX11* in NOA39 and NOA49. **A** The families affected by the variants in *TEX11*. The red dotted line indicates mutated positions in the Sanger sequencing results. **B** Testicular histopathology. **C** The mutated positions of *TEX11* are conserved among species (red arrows). And the dotted lines indicate the positions of *TEX11* variants in TEX11 protein. M, mutation; WT, wild type

**Table 1:** Deleterious variants detected in patients with non-obstructive azoospermia and related clinical phenotypes.

Individual	NOA16	NOA50	NOA51	NOA22	NOA25	NOA42	NOA39	NOA49
**Gene**	*FANCA*	*FANCA*	*SYCE1*	*DMRT1*	*DMRT1*	*PLK4*	*TEX11*	*TEX11*
**Inheritance pattern**	AR	AR	AR	AD	AD	AD	X-linked	X-linked
**RefSeq accession number**	NM_000135	NM_000135	NM_001143763	NM_021951	NM_021951	NM_001190799	NM_031276	NM_031276
**Age**	27	27	31	27	31	29	32	25
**Secondary sexual characteristics**	Normal	Normal	Normal	Normal	Normal	Normal	Normal	Normal
**testicular volume (Left/Right, ml)**	8/8	8/8	15/15	10/10	10/10	12/12	12/12	10/10
**Somatic karyotype**	46,XY	46,XY	46,XY	46,XY	46,XY	46,XY	46,XY	46,XY
**Y Chromosome microdeletions**	No	No	No	No	No	No	No	No
**Follicle-stimulating hormone (IU/L)**	23.87	24.74	3.85	16.32	26.54	29.24	8.44	4.02
**Luteinizing hormone (IU/L)**	6.10	9.38	0.41	6.44	11.35	7.05	6.33	5.33
**Testosterone (nmol/L)**	14.03	9.64	31.14	17.95	7.07	10.75	10.75	13.34
**Estradiol (pmol/L)**	NA	90	372	241	23	73	97	132
**Prolactin (ng/ml)**	NA	8.26	14.6	11.88	10.37	8.11	8.92	10.24
**Testis histology**	SCOS	ND	MA	SCOS	SCOS	SCOS	Hypospermatogenesis	MA
**Hom/Het**	Hom	Het/ Het	Hom	Het	Het	Het	Hemi	Hemi
**cDNA mutation**	c.3263C>T	c.3263C>T/ c.1729C>G	c.689_690del	c.425C>T	c.340G>A	c.2785A>G	c.466A>G	c.559_560del
**Mutation type**	Missence	Missence/ Missense	Frameshift	Missense	Missense	Missense	Missense	Frameshift
**Protein alteration**	p.S1088F	p.S1088F/ p.P577A	p.F230fs	p.A142V	p.V114M	p.M929V	p.M156V	p.M187fs
**1KGP**	0.0218	0.0218/ 0	0	0	0	0	0	0
**EXAC_EAS**	0.0235	0.0235/ 0	0	0	0	0	0.0039	0
**gnomAD_EAS**	0.0265	0.0265/ 0	0.0001	0	0	0	0.0034	0
**SIFT**	D	D/ D	NA	T	D	D	T	NA
**PolyPhen-2**	P	P/ D	NA	D	D	P	B	NA
**MutationTaster**	N	N/ D	NA	D	D	D	N	NA
**CADD**	21.8	21.8/ 23.9	NA	22.2	33	23.9	22.2	NA
**HGMD**	NA	NA/ NA	NA	NA	NA	NA	D	NA
**Validation in patient**	Yes	Yes/ Yes	Yes	Yes	Yes	Yes	Yes	Yes
**Mother/Father genotype**	Het/Het	ND/ ND	ND/Het	ND	ND	ND	Het/WT	ND

*AR* autosomal recessive, *AD* autosomal dominant, *1KGP* 1000 Genomes Project, *ExAc_EAS* the data of East Asian in Exome Aggregation Consortium, *gnomAD_EAS* the data of East Asian in the Genome Aggregation Database, *D* Damaging, *T* Tolerant, *P* Possibly Damaging, *B* Benign, *N* Polymorphism, *ND* Not Detect, *SCOS* Sertoli cell only syndrome, *MA* maturation arrest

The original article [1] has been corrected.

## References

[1] Tang D, Li K, Geng H (2022). Identification of deleterious variants in patients with male infertility due to idiopathic non-obstructive azoospermia. Reprod Biol Endocrinol.

